# Potential Implications of Citrulline and Quercetin on Gut Functioning of Monogastric Animals and Humans: A Comprehensive Review

**DOI:** 10.3390/nu13113782

**Published:** 2021-10-25

**Authors:** Victoria Anthony Uyanga, Felix Kwame Amevor, Min Liu, Zhifu Cui, Xiaoling Zhao, Hai Lin

**Affiliations:** 1 Shandong Provincial Key Laboratory of Animal Biotechnology and Disease Control, Department of Animal Science, College of Animal Science and Veterinary Medicine, Shandong Agricultural University, Tai’an City 271018, China; uyangava@sdau.edu.cn (V.A.U.); lmliumin1991@sina.com (M.L.); 2 Organization of African Academic Doctors (OAAD), Off Kamiti Road, Nairobi P.O. Box 25305-00100, Kenya; amevorfelix@gmail.com; 3 Farm Animal Genetic Resources Exploration and Innovation Key Laboratory of Sichuan Province, Sichuan Agricultural University, Chengdu 611130, China; 2018102013@stu.sicau.edu.cn

**Keywords:** tight junctions, flavonoids, intestinal immunity, metabolism, nitric oxide, microbiota

## Abstract

The importance of gut health in animal welfare and wellbeing is undisputable. The intestinal microbiota plays an essential role in the metabolic, nutritional, physiological, and immunological processes of animals. Therefore, the rapid development of dietary supplements to improve gut functions and homeostasis is imminent. Recent studies have uncovered the beneficial effects of dietary supplements on the immune response, microbiota, gut homeostasis, and intestinal health. The application of citrulline (a functional gut biomarker) and quercetin (a known potent flavonoid) to promote gut functions has gained considerable interest as both bioactive substances possess anti-inflammatory, anti-oxidative, and immunomodulatory properties. Research has demonstrated that both citrulline and quercetin can mediate gut activities by combating disruptions to the intestinal integrity and alterations to the gut microbiota. In addition, citrulline and quercetin play crucial roles in maintaining intestinal immune tolerance and gut health. However, the synergistic benefits which these dietary supplements (citrulline and quercetin) may afford to simultaneously promote gut functions remain to be explored. Therefore, this review summarizes the modulatory effects of citrulline and quercetin on the intestinal integrity and gut microbiota, and further expounds on their potential synergistic roles to attenuate intestinal inflammation and promote gut health.

## 1. Introduction

Nutrition directly influences gut microbial composition and functions, with significant impacts on host health [1]. The microbiota, dietary factors, and their metabolites are in close contact to the gut epithelium, forming a thin cell-layer that separates the constituents of the host from its external setting [2,3]. Generally, the tight junction proteins (including claudins, zona occludin-1, and occludin), act to maintain the gut barrier integrity, in turn promoting epithelial cell barrier functions [4]. It is obvious that during gut disorders, the functionality of the tight junction proteins declines, which would lead to the gut becoming permeable to toxic substances, causing severe systemic inflammatory responses [3]. Therefore, changes in the gut microbiota may produce disorders including cancer, leaky guts, diabetes, obesity, and neurological disorders [5]. Inflammatory bowel disease (IBD) occurs due to dysregulation in the immune system, leading to intestinal inflammation and microbial dysbiosis [3]. Studies have reported on the roles of dietary supplements, and their effects on the microbiota and gut metabolites during physiological processes such as immunity, metabolism, and neurological and nutritional homeostasis [6,7]. Moreover, nutritional strategies intended at repairing intestinal mucosal damage also tend to elicit beneficial effects on the microbiota, immune system, digestive physiology, and inflammatory response [5]. Metagenomic analyses have revealed amino acid metabolism and transport as an integral metabolic activity of the intestinal microbiota [8]. This suggests that the positive benefits of nutritional intervention would not only modulate gut function, but ameliorate the adverse impacts of gut inflammation and dysfunction under various pathophysiological conditions.

Citrulline (CIT) (commonly found as l–citrulline), a non-essential amino acid with unique metabolic properties, has emerged as a promising pharmaconutrient [9]. Citrulline has been implicated in several regulatory roles, including gut modulation, antioxidative and anti-inflammatory effects, protein synthesis, nitrogen homeostasis, blood pressure regulation, renal function, cardiac function, skeletal muscle function, vascular health, lipid and energy metabolism, arginine production, and thermoregulation [10,11,12,13]. Several studies have revealed the efficacy of citrulline in serving as an arginine precursor and in nitric oxide production, especially in cases of acute or chronic inflammation [14,15]. Relatively few studies are available on citrulline in animals; however, it is subtly gaining research interest due to its unique metabolism. Citrulline is a functional marker of gut barrier dysfunction [16], and has been associated with various intestinal diseases, including short bowel syndrome [17,18], gastric ulcers [19], and necrotizing enterocolitis [20]. Recently, flavonoids, a group of natural compounds abundant in plants sources, such as bark, roots, stems, fruits, vegetables, bulbs, wine, and tea, have gained widespread attention for their therapeutic effects such as anti-oxidative, anti-inflammatory, cardioprotective, anti-estrogenic, neuroprotective, and chemoprotective roles [21,22]. Quercetin is the primary polyphenolic flavonoid present in foods and it has been studied for its numerous beneficial properties including enhancing gut microbial composition, functions, and disease prevention [2].

This review aims to expound our understanding on the functional roles of citrulline and quercetin on gut health, their interaction with the gut microbiota, and their actions to establish gut immune homeostasis. We further discuss the potential synergistic roles for which they can be utilized, as this currently exists as a huge knowledge gap. An understanding of the roles exerted by citrulline and quercetin to mediate gut functions either individually or when co-utilized would expand the knowledge base for the application of these dietary supplements in health and disease conditions.

## 2. Overview on Citrulline and Quercetin

**Figure 1:**
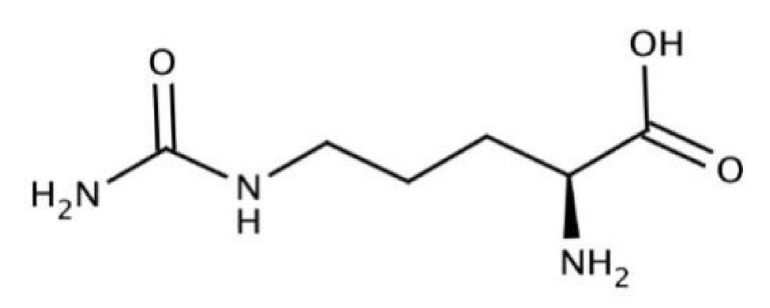
Chemical structure of l-citrulline.

l-citrulline, C_6_H_13_O_3_N_3_, is a non-protein, neutral, non-essential, alpha-amino acid that also serves as an important metabolite of the urea cycle [23]. l-citrulline has an alpha-structured chemical configuration (Figure 1), with a molecular mass of 175.19 Daltons, and high solubility in water [24]. As a non-protein monomer, citrulline does not undergo protein synthesis but rather can occur from post-translational modifications and products of the Golgi body [24]. Naturally, citrulline is largely concentrated in watermelon (*Citrullus lanatus* (Thunb.) Matsum. & *Nakai Cucurbitaceae*) fruits, but although present in vegetative tissues (such as stems, seedlings, and leaves) and root, citrulline is confirmed to progressively accumulate majorly in the fruit flesh and rind tissues of watermelons [25]. It can range from 1.6 to 3.5 g/kg l-citrulline in freshly obtained watermelon [11].

**Figure 2:**
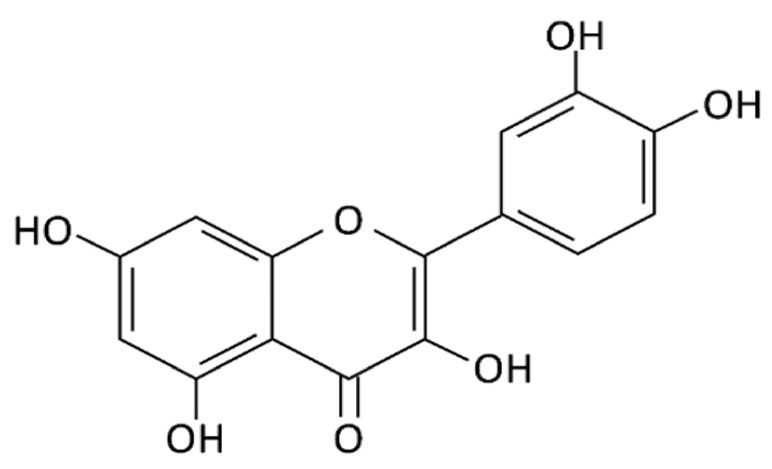
Chemical structure of quercetin.

Quercetin (3,3′,4′,5,7-pentahydroxyflavanone) exists in several forms as quercetin glycoside, quercetin sulfate, quercetin glucuronide, and methylated quercetin [26]. Biochemically, quercetin is an aglycone that contains no sugar molecule and shows partial solubility and insolubility in hot water and cold water, respectively. It is, however, completely soluble in lipids and alcohol [26,27]. The formation of quercetin glycoside is through the attachment of quercetin to a sugar group (Figure 2). The resulting structure after the successful attachment has a changed biochemical structure which allows for easy solubility, absorption, and metabolism [27]. Research has recently focused on the application of quercetin to promote the composition of the gut microflora, facilitating immunoregulation and gut functions [28]. Quercetin can be extracted from various plants including *Moringa oleifera, Sophora Japonica* L., berries, apples, and onions [28,29]. Quercetin exhibits unique biological characteristics including antibiotic, anti-oxidative, anti-inflammatory, immunomodulatory, and anti-fibrotic effects [26,30,31]. As an important dietary antioxidant, quercetin scavenges reactive oxygen species (ROS) and reactive nitrogen species (RNS), inhibits lipid oxidation, and chelates iron, protecting cells against oxidative damages [32].

## 3. Metabolism of Citrulline and Quercetin

### 3.1. Metabolism of l-Citrulline

l-citrulline is essentially biosynthesized through the nitric oxide and the urea cycle, with the urea cycle accounting for ~90% production, and the nitric oxide cycle producing ~10% of l-citrulline [24,33]. Citrulline can be directly formed from l-arginine via the activity of nitric oxide synthase (NOS) enzymes, which exist as constitutive (cNOS) and inducible (iNOS) isoforms [34]. The cNOS is calcium/calmodulin-dependent and found in endothelial cells (eNOS) and neuronal cells (nNOS), whereas, the iNOS, which is calcium/calmodulin-independent, is triggered during inflammatory conditions by bacterial toxins or cytokines in many cell types, including macrophages, neutrophils, and endothelial cells [35].

**Figure 3:**
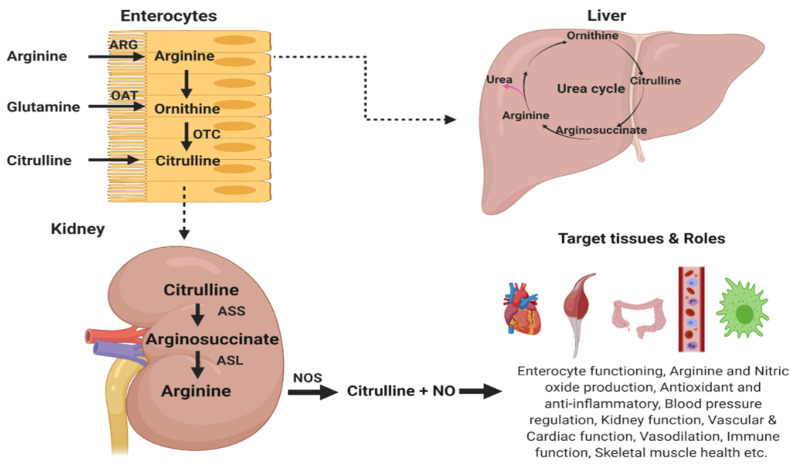
Overview of citrulline metabolism and related amino acids. ARG, arginase; OAT, ornithine aminotransferase; OCT, ornithine carbamoyltransferase; ASS, argininosuccinate synthetase; ASL, argininosuccinate lyase. Created with BioRender.com, accessed on 6 February 2021.

Within the small intestine, citrulline is synthesized from glutamine by the enterocytes, releasing citrulline into blood circulation for metabolism into arginine by the kidneys [36]. The small intestine has been established as the major site for citrulline production due to the abundance of citrulline synthesizing enzyme (pyrroline-5-carboxylate synthase, P5CS) and the lower activity of citrulline catabolizing enzymes, such as argininosuccinate synthase and argininosuccinate lyase [9,37]. In citrulline synthesis from glutamine, intestinal catabolism plays a significant role in humans, pigs, and rats, since the enzyme intermediate P5CS, is almost exclusively located in the intestinal mucosa [38,39]. l-citrulline synthesis from l-glutamine occurs through a transaminase reaction in the enterocytes [11]. The citrulline produced from the enterocytes is absorbed by the kidney (proximal tubular cells), and the enzymatic actions of arginosuccinate synthase and arginosuccinate lyase rapidly convert the l-citrulline formed into l-arginine [38] (Figure 3). This reaction pathway provides ~50% of daily arginine needs in young mammals [39], and meets the entire arginine requirement in healthy adult mammals [40]. Using irinotecan-treated rats, mRNA expression analysis revealed that P5CS, glutaminase, ornithine aminotransferase, and ornithine carbamoyltransferase enzymes, which are responsible for citrulline synthesis from glutamine in enterocytes, were downregulated, whereas arginase 2 and proline dehydrogenase, responsible for citrulline synthesis from proline or arginine, were unaffected [41].

Citrulline serves as the direct precursor of arginine (ARG), an amino acid that is involved in several physiological roles including urea cycle function, protein synthesis, creatine and polyamines synthesis, ammonia detoxification, and nitric oxide (NO) synthesis [38], although its priority for utilization under different conditions remains puzzling [40]. Within the intestine and liver, arginine catabolism is increased, largely due to higher arginase expression, subjecting ingested arginine to the first-pass extraction. Contrastingly, l-citrulline bypasses extraction by the gastrointestinal tract (GIT) and liver, promoting the down-stream synthesis of NO via l-citrulline recycling to l-arginine [11]. Studies have shown that augmenting l-arginine levels as a potential therapeutic mechanism in conditions of NO unavailability/deficiencies may be largely ineffective, since l-arginine is subject to gastrointestinal and hepatic extraction, along with a dose-dependent effect of gastrointestinal distress [11]. Alternatively, the compartmentalization of hepatic l-citrulline metabolism in the urea cycle permits l-citrulline to bypass hepatic catabolism, thus allowing for l-citrulline supplementation to effectively increase the peripheral levels and tissue contents for l-arginine and NO [11,23]. In mice, citrulline supplementation is more efficacious at increasing arginine availability (by 35%) compared to direct arginine supplementation, since its only fate is conversion to arginine in vivo [42]. Extensive arginine catabolism occurs in the small intestinal mucosa, with proportions of arginine being removed via splanchnic first-pass metabolism (FPM). This catabolism engulfs about 40% of the luminal arginine in adult rats, 38% of dietary arginine in adult humans [39], and ~70% in mice with limited quantities entering into peripheral circulation [42], suggesting that significant quantities of supplemental arginine are unavailable to extra-intestinal tissues [39]. Additionally, exogenous l-citrulline administration elevated serum-free contents of amino acids including arginine, citrulline, and ornithine in a dose-dependent manner [43].

The ARG–CIT–ARG inter-organ cycle is a machinery that protects dietary arginine from excessive hepatic degradation (Figure 3), thus maintaining protein homeostasis [44]. Further evidence showed that, during conditions of arginase-induced arginine deficiency, it was l-citrulline supplementation that increased NO concentration and microcirculation in tissues, not L-arginine [45,46]. The combination of l-citrulline plus l-arginine produces rapid kinetics in enhancing NO-dependent responses and plasma arginine, greater than arginine alone [47]. Thus, for conditions associated with impaired arginine metabolism, arginine deficiencies and NO metabolism disorders, l-citrulline administration offers a potential therapeutic strategy [23].

### 3.2. Metabolism of Quercetin

The pathways through which quercetin is absorbed in the gut of both humans and animals are well documented [48,49]. Absorption of quercetin mostly occurs in the small intestine. Thereafter, it is metabolized by the animal’s body. However, due to the difficulty in digesting quercetin in the small intestine, it may undergo further catabolism in the colon initiated by the intestinal microflora to produce bioavailable compounds that are easily absorbed in the form of conjugates with attached groups of sulfate, methyl, or glucuronide [48,49]. Although the main site for quercetin absorption is the small intestine [50], about 5 to 10% of it undergoes complete absorption in the small intestine, whereas, about 90 to 95% of quercetin is absorbed in the colon [51,52,53]. This occurrence is attributed to the fact that glucose groups are attached to quercetin and before quercetin can be absorbed (aglycone, the absorbed unit of quercetin which is very reactive and insoluble in aqueous solution) [54,55], into the enterocyte, the attached sugar molecules or any other chemical substances must be removed, usually by the activities of the brush border enzymes including lactase phloridzin hydrolase (LPH) which removes sugar groups from flavonoids [56]. In comparative terms, the bioavailability of quercetin glycosides is more than quercetin aglycone, because of the insolubility of aglycone in the gut lumen [57,58,59]. Since the enzymes in the brush border are more glucose-specific, the absorbability of quercetin glucosides is rapid compared to other forms of glycosides such as rutin (quercetin-3-*O*-rutinoside) which, via the actions of enzymes from the intestinal microflora, undergoes deglycosylation to form aglycone [60,61,62,63]. Literature has outlined the significance of solubility on quercetin bioavailability in several animal models (mice, rats, humans, pigs, and chickens), stating clearly that the solubility and bioavailability of quercetin can be improved when combined with substances such as alcohol [64], nondigestible oligosaccharides [65], or with a high-fat (17%) diet [66,67]. When quercetin is absorbed into the enterocytes, it is glucuronidated, sulfated, and methylated by UDP-glucuronosyl transferases (UGTs), sulfotransferases (SULTs), and catechol-*O*-methyl transferase (COMT), respectively, inhabited in the gut and hepatic cells. This conversion reaction was also confirmed by an in vitro experiment using hepatocytes obtained from rats or humans [68]. The absorption of quercetin is proceeded by its appearance in different biochemical forms in the bloodstream, such as its methylated form. Studies have evaluated the various species of quercetin in blood plasma and reported that conjugates of quercetin represented 78 to 79%, with 8.5 to 11% existing as isorhamnetin (3′-*O*-methyl-quercetin), and 10 to 13% as tamarixetin (4′-*O*-methyl-quercetin) conjugates [61,62,67]. Reports indicated that enterocytes, via multidrug resistance-associated protein 2, help to excrete a substantial percentage of flavonoid conjugates back into the intestinal lumen [69]. Generally, quercetin glucuronides are in a more stable state for transportation into the bloodstream. However, when they are transported into the vascular smooth muscle cells [70,71] and inflammation sites, for instance, they may undergo deconjugation [71,72,73]. Quercetin glucuronide itself exhibits some diminishing activities in comparison to aglycone, and occasionally the conjugated and/or methylated compounds exhibit some unique bioactivities different from that displayed by the parent compound [74,75,76,77,78].

**Figure 4:**
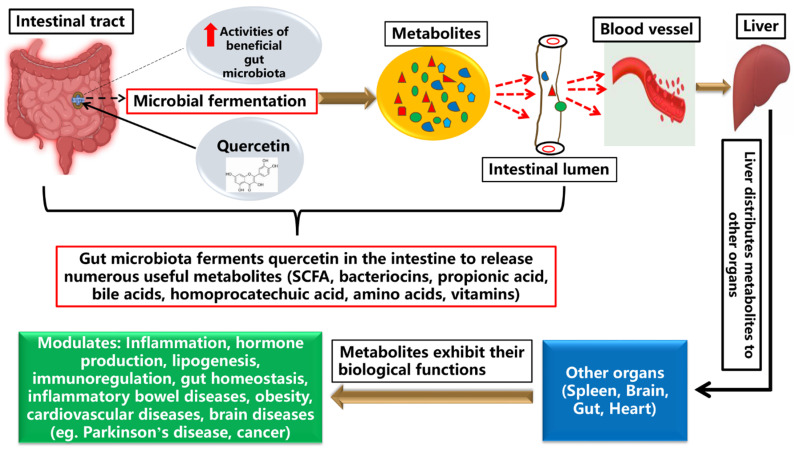
Quercetin is fermented into several metabolites in the gut by numerous gut microbial species. These metabolites are useful substances that are diffused into the blood stream to be distributed to the various organs where their biological activities are exhibited.

Some derivatives of quercetin such as rutin cannot be absorbed in the small intestine and so it goes through deglycosylation in the colon by some gut enzymes (β-glucosidases and α-rhamnosidases) produced by the intestinal microflora. At this site (the colon), some of the deglycosylated rutin will be absorbed and others will undergo gut microbial catabolism into smaller soluble and absorbable molecules [79]. The aglycone then produced will be absorbed by the colonocytes and then transported into the bloodstream or may undergo further gut microbial breakdown to form simpler and more soluble species for easy absorption. Certain strains of gut microbiota including *Bacteroides* spp., *Streptococcus* spp., *Bifidobacterium* spp., *Pediococcus* spp., and *Lactobacillus* spp. are involved in the transformation of quercetin into various phenolic acids including 3,4-dihydroxyphenylacetic, 3-hydroxybenzoic, and 3,4-dihydroxybenzoic acids [80]. Other in vitro reports showed that the contents of porcine hindgut could metabolize quercetin into several species [69]. An in vitro experiment on quercetin fermentation in the colon showed that protocatechuic acid was the main product obtained, whereas *p*-hydroxybenzoic, homovanilic, and phenylacetic acids were also observed but in smaller quantities [81]. Similar products were obtained after exposing quercetin to exhaustive electrochemical hydrolysis [81,82]. In a rat model, the fermentation of quercetin by the intestinal microflora produced important biological acids including propionic acid (3-(3,4-dihydroxyphenyl)) which was further degraded to form 3,4-dihydroxyphenylacetic acid, and, if necessary, could be further fermented to form protocatechuic acid and subsequently 4-hydroxybenzoic acid [83]. The catabolism process continues until simpler forms are obtained and then lastly produces carbon dioxide [83,84]. The metabolic pathways of quercetin metabolism in monogastric and human studies are depicted in Figure 4.

## 4. Role of Citrulline and Quercetin in Gut Functioning

### 4.1. Effects of Citrulline on Gut Functions of Animals

Studies have shown the beneficial effects of increased dietary levels of amino acids on gut development, functions, and immune defense of animals during normal conditions and periods of intestinal challenge [85]. Citrulline has been identified as a gut metabolite, and although it is a non-protein amino acid, it can be synthesized from several amino acids in the enterocytes [86]. Circulating citrulline levels can serve as biological markers in the assessment of mucosal damage. For instance, the loss of small bowel epithelial cell mass is reflected by lowered circulating citrulline levels [87]. There exists an association between plasma citrulline and intestinal enterocyte mass, as well as the small bowel remnant length. Therefore, plasma citrulline derived from the small intestine can depict intestinal failure independent of the nutritional and inflammatory state [36,88]. Plasma citrulline had been shown to relate positively with small intestinal absorptive capacity [89,90], and negatively with the extent of mucosal damage [91]. In addition, the relative decrease in plasma citrulline correlated with pathological findings of crypt necrosis, villus atrophy, and enterocyte loss [92]. Therefore, citrulline is widely considered as a blood biomarker for gastrointestinal functioning [36].

Citrulline concentration depicts overall small bowel functioning and provides reliable information on global gut absorption [37]. Citrulline pretreatment improved gut barrier integrity and reduced bacterial translocation in ileum mucosa [13]. It serves as an indicator for early acute intestinal dysfunction, such that reduction in the number and functions of intestinal epithelial cells would result in decreased serum citrulline levels [93]. Moreover, significant correlations have been established between plasma citrulline concentration and small bowel length, and villous atrophy [36]. Citrulline also exhibited protective effects against inflammatory changes and intestinal permeability, by improving the expression of tight junction proteins (occludin and zona occludins-1) during early-staged dietary-induced non-alcoholic liver disease (NAFLD) in mice [94].

The gut microbiota is critically involved in the pathogenesis of immune diseases. It influences the host’s innate immune response through alterations in immunological mediators, such as cytokines and chemokines [95]. In understanding gut microbiota dysbiosis, alterations in enterocyte function, identified with lowered blood citrulline, was associated with increased abundance of *Flavobacteriaceae* and decreased abundance of *Streptococcaceae* and *Lachnospiraceae* [96]. This further indicated the existing linkage between enterocyte functioning and gut microbiota in the development of metabolic complications. Joint analysis of the microbiome, intestinal metabolites, and hepatic mRNA sequence showed a highly positive association (|correlation| > 0.9) between primary bacteria (*Lactobacillus, Bifidobacterium*, *Streptococcus*, *Ruminococcaceae*, *Oscillospira*, *Peptococcaceae*, *Roseburia*, *RF39*, *Turicibacter*, and *Veillonella)*, key hepatic genes, and major metabolites including citrulline, histidine, isoleucine, guanine, deoxycholic acid, galacturonic acid, and glucuronic acid [97]. Ho, El-Nezami, and Shah [98] demonstrated the synergistic effect of citrulline and *Lactobacillus helveticus* strain in promoting intestinal epithelial barrier functions, revealing the prebiotic properties of citrulline.

As an amino acid that is highly abundant in watermelon [99], supplementation of watermelon products altered the cecal microbiome in high-fat-diet (HFD)-fed mice causing distinct shifts in the relative abundance of *Bacteroides* genus, and a decrease in *Clostridiales* bacteria, and family *Ruminococcaceae* with the intake of watermelon products [100]. In HFD-fed mice, treatment with watermelon products (fiber, rind, and seed) significantly elevated the hepatic citrulline levels, especially using the watermelon seed, and further influenced metabolites involved in lipid metabolism by reducing long-chain FA (LCFA) stearate, bile acids contents (cholate, β-muricholate, and tauro-β muricholate), and arachidonic acid derivatives (12-hydroxyeicosatetraenoic acid (12-HETE) and 15-HETE). This implied an enterohepatic recirculation and anti-inflammatory role [100]. In probiotic-fed groups, dietary supplementation with *Lactococcus lactis* WFLU12 greatly influenced metabolic pathways by increasing citrulline production to promote growth regulation and gut development in fish [101]. Fecal metabolomics of type 2 diabetic nephropathy (T2DN) rats identified citrulline as a metabolic marker involved in the urea cycle and closely implicated in the improvement of T2DN, protecting against inflammation and renal damage [102]. Furthermore, to investigate the adverse impact of phthalate-related disruption in zebrafish, it was discovered that microbial metabolites including the citrulline levels were lowered, which may have had additive effects on the immune cells and enterocyte functioning [103]. Table 1 gives a summary of studies that demonstrated the functional roles of citrulline in modulating intestinal immunity and gut health.

### 4.2. Effects of Quercetin on Gut Functions of Animals

Quercetin enhances intestinal barrier function and modulates gut microbiota composition. The activities of tight junction proteins at the gut epithelium enhance barrier functions and reduce inflammation, protecting the host from colonic diseases [115]. Quercetin promotes the assembly of tight junction proteins such as ZO-2, occludin, claudin-1, and claudin-4 expression by inhibiting the PKCδ isoform [115]. Quercetin induced the remodeling of epithelial tight junctions and enhanced barrier integrity in the Caco-2 gastrointestinal epithelial cell model [116]. Studies have directly employed the use of specific metabolites produced by gut bacteria (such as *Bifidobacterium pseudocatenulatum INIA P815* strain [3], *Lactobacillus*, *Clostridium*, and *Bacteroides* [4]) from flavonoids, including quercetin, as therapeutic agents in treating diseases such as inflammatory bowel disease (IBD). It was shown that Urolithin A, UAS03, and L-tryptophan mitigated IBDs by increasing epithelial cell junction proteins in the gut, and reduced gut inflammation [3,4]. These metabolites did not only act to reduce inflammation but also restored gut barrier integrity and protected against colitis [3,4]. Wu et al. (2019) outlined that L-tryptophan, a metabolite obtained from quercetin metabolism, serves as a nutrient enhancer, as well as in the regulation of the kynurenine pathway and immune responses in the mice model.

**Figure 5:**
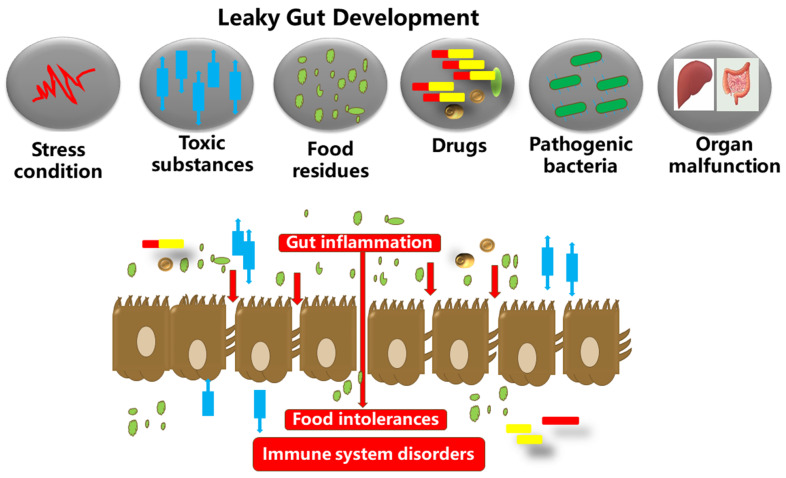
The gut microbiota, food substances, and the gut epithelium are in proximity. Dysbiosis in the gut microbiota could inversely increase the gut permeability. This results in autoimmune and neurodegenerative disorders, and excessive inflammation in the gut epithelium, which in turn degenerates the tight junction proteins and membranes (occludin, claudins, junctional adhesion molecules, and tri-cellulin, ZO-1, and cingulin). The tight junction barriers are disrupted by the invasion of toxic substances (bacterial endotoxins, metabolites, food particles, drugs, etc.), resulting in barrier dysfunction and severe inflammatory disorders such as IBDs.

Quercetin is transformed by the gut microbes (*B. fragilis*, *Lactobacillus L-2*, *C. perfringens*, *E. ramulus*, *Bifidobacterium B-9*, *Streptococcus S-2*, and *Bacteroides* JY-6) into several metabolites including 3,4-dihydroxybenzoic acid, 4-hydroxybenzoic acid, 3,4-dihydroxyphenylacetic acid (homoprocatechuic acid), and 3-(3-hydroxyphenyl) propionic acid [79]. The 3,4-dihydroxyphenylacetic acid was reported as an active flavonoid metabolite that prevents liver damage by upregulating transcription factor nuclear factor (erythroid-derived 2)-like 2 (Nrf2) [117], and also exhibited a strong anxiolytic effect in mice model [118]. However, 3-(3-hydroxyphenyl) propionic acid showed a significant vasodilatory effect in rats, by modulating the endothelial nitric oxide synthase (eNOS)-derived nitric oxide (NO) [111]. Lastly, 4-hydroxybenzoic acid was also reported to be involved in the scavenging activities of free radicals and inhibition of trypsin activity [119]. Quercetin can reshape gut microbiota to alleviate several diseases [28]. In antibiotic-treated mice, quercetin supplementation improved the diversity of the gut bacterial community, increased the length of intestinal villi, and improved mucosal thickness [120]. Another study also reported that quercetin suppressed the production of pro-inflammatory cytokines, such as interleukin (IL)-17, TNF-α, and IL-6 in the colon tissues, and enhanced the population of *Bacteroides*, *Bifidobacterium*, *Lactobacillus*, and *Clostridia*, but significantly reduced those of *Fusobacterium* and *Enterococcus* [121]. Quercetin also inhibited the growth of *Porphyromonas gingivalis*, *Actinomyces viscosus*, *Fusobacterium nucleatum*, *Actinomyces naeslundii*, and *Helicobacter pylori* [121]. Moreover, it has been proposed that quercetin similarly affects the gut microbiota as tea catechins and other flavonoids [122]. However, further studies are necessary to expound on this (Figure 5).

Reports indicated that supplementing quercetin in a high-fat-diet-fed mice model modulated gut microbiota composition via lessening the activation of the lipoperoxidation-dependent TLR-4 pathway [123]. Another study confirmed the prebiotic role of quercetin as it reduced the abundance of atherogenic-related bacteria (*Verrocomicrobia*) and the abundance of atherogenic lipid metabolites but increased the abundance of *Firmicutes*, *Cyanobacteria*, and *Actinobacteria* [124]. Quercetin increased the diversity of microbiota inhabiting the colon of mice infected with *Citrobacter rodentium*, hence lessening colitis and decreasing pro-inflammatory cytokines [125]. Recently, it was demonstrated that quercetin improved gut dysbiosis in antibiotic-treated mice by successfully increasing the diversity of the gut microbiota, and restored barrier functions through decreased expressions of serum D-lactic acid and serum diamine oxidase activity [110]. Additionally, quercetin supplementation increased the relative abundance of *Lactobacillus* in the cecum, and the expression of tight junction proteins (mucin-2 and tight junction protein 1) was downregulated [121]. Cumulatively, these reports provide evidence for quercetin’s role in improving gut barrier functions, preventing inflammatory bowel diseases, and promoting the composition of beneficial microbiota (Table 2).

### 4.3. Potential Combinatory Roles for Citrulline and Quercetin in Gastrointestinal Health of Animals

**Figure 6:**
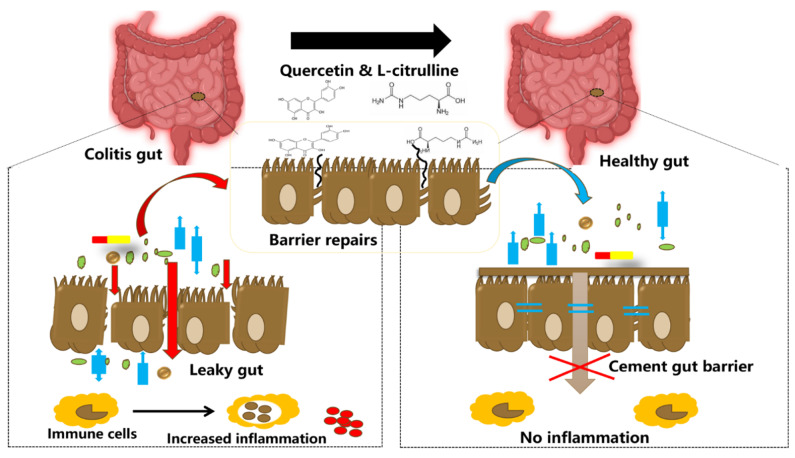
Illustration showing tightening of the gut barrier cells and reduced inflammation due to the introduction of quercetin and l-citrulline.

In the following section, we discuss the potential of utilizing both l-citrulline and quercetin together on specific aspects of gut health and immunity. The gastrointestinal tract environment is exposed to several commensal bacteria, and dietary modifiers, such as, probiotics, prebiotics, symbiotic, polyphenols, amino acids, and other bioactive compounds, which have been demonstrated to beneficially impact gut health and host welfare [133]. Gut health is principally affected by two important factors, the intestinal barrier and gut microbiota [133]. Tight junctions comprise of transmembrane proteins (such as occludin, claudins, junctional adhesion molecules, and tri-cellulin) and peripheral membrane proteins (ZO-1 and cingulin), which function to regulate paracellular permeability and gut barrier integrity [3]. Tight junction disruption leads to barrier dysfunction and this is implicated in the pathology of inflammatory bowel diseases (IBDs) [121] (Figure 5). Specifically, gut barrier dysfunction allows for bacterial invasion and excessive inflammation in the gut [3]. The release of inflammatory cytokines and growth factors such as interferon-γ (IFN-γ), TNF-α, IL-1β, and TGF-α, platelet-derived growth factors, and bacterial endotoxins, further alters the tight junctions, increasing permeability [3]. Dysbiosis in the gut microbiota increases gut permeability (preferably called leaky gut) and significantly paves way for the pathogenesis of several disorders, such as autoimmune and neurodegenerative disorders [125,134]. As such, the leaky gut is mainly caused by excessive inflammation in the gut epithelium, which in turn degenerates the tight junction proteins [3]. On this note, it is evident that barrier dysfunction and inflammation are the main inter-correlated conditions that permit the occurrence of IBDs, and hence, alleviating these conditions is crucial for mitigating the disease progression (Figure 6). Noteworthy is the fact that IBD pathogenesis is influenced by several contributing factors, including environmental, genetic, inflammatory factors, oxidative stress, and intestinal microbiota [125,134].

This development has prompted research on several nutritional interventions including plant-based flavonoids, organic/inorganic metabolites [3], and amino acids that displayed potent anti-inflammatory, gut-protective, and antioxidative properties [4]. Additionally, there exists crosstalk between the gut microbiota and enterocytes that shape the gut environment, which intensely affects the intestinal immune homeostasis [3,88]. Changes in the gut microbiota can modulate the host’s metabolic phenotype and immune status, and in turn, the immune system shapes the composition of the gut microbiota [88].

(a) Anti-inflammatory and immunomodulatory functions

Plasma citrulline level has been extensively adopted in research to establish its role as a reliable and quantitative biomarker of intestinal diseases. Citrulline has been demonstrated as efficacious in IBD models typically induced as Crohn’s disease or ulcerative colitis [88]. Using an ulcerative colitis model in rats, citrulline supplementation elicited protective effects in restoring body weight, and lowered the histopathology score and pro-inflammatory factors (MCP-1, IL-6, IL-17A, and p-STAT3) in colon tissues, possibly by improving the structural integrity and absorptive function of the intestine [135]. Citrulline is a reliable marker of intestinal malabsorption [136], and besides its role as a marker of enterocyte function, citrulline has been found to reflect gut dysbiosis induced by antibiotics [137]. l–citrulline alleviated the gastric mucosal lesions and inhibited malondialdehyde (MDA) and myeloperoxidase (MPO) activity during ischemia-reperfusion in rats, thus eliciting protective effects on gastric mucus synthesis and secretion [113]. Further, plasma citrulline levels are elevated in multiple sclerosis [138]. Thus, citrulline is a validated marker of intestinal barrier functions and disorders [13,37].

Studies in immunonutrition have targeted arginine supply, considering its essential role as a substrate for NO production by macrophages [14]. Interestingly, reports showed that citrulline has the potential to act as an immunonutrient to modulate host defense. In vitro and in vivo investigations showed that citrulline may directly influence macrophage function and modify NO production [139]. During mycobacterial infections, T cells can metabolize l-citrulline to replenish intracellular l-arginine to maintain cellular proliferation, cytokine production, and inflammatory function [140]. In diabetic obese rats, the associated macrophage dysfunction was attenuated with citrulline administration to initiate NO production and regulation of TNF-α and IL-6 cytokines [141]. In infantile rats, both l-arginine and l-citrulline modulated regulatory T-cell functions [142]. Plasma citrulline further reflects on acute intestinal impairment, since it decreases at the onset of digestive bacterial translocation and septic shock [143]. A study reported a lowered number of inflammatory biomarkers following citrulline supplementation [144]. It acted as an anti-inflammatory agent, characterized by reduction in pro-inflammatory cytokines, along with increase in anti-inflammatory cytokines. l-citrulline exerted protective effects by reducing IL-1β and IL-12, but increased IL-10 generation [145]. Citrulline has been shown to induce anti-inflammatory cytokines (IL-10 and TGFβ), while downregulating the pro-inflammatory (IL-1β and MMP9) genes related to leucocyte migration [146]. In ulcerated rats, citrulline pre-treatment attenuated the elevation in IL-6, iNOS, and MPO activities during ethanol challenge [19]. Citrulline was inversely correlated with biomarkers of systemic inflammation, such as C-reactive protein and ferritin concentrations [147].

It is well known that quercetin interacts with other dietary components, such as selenium, polyunsaturated fatty acids, sulfur-containing amino acids, minerals, and other antioxidants including resveratrol [28] to improve gut function and immunity in animals [2]. Quercetin has the potential to confer cell-mediated immunity via downregulation of inflammatory cytokines gene expression [148]. In heterophils, quercetin showed anti-inflammatory function by suppressing pro-inflammatory cytokines and several ROS-related gene expressions to combat fungal infections [148]. Dietary quercetin supplementation improved the secretion of immunoglobulins (IgA and IgM), and cytokines networks including IL-4, TNF-α, TRAF2, and TNFRSF1B [149]. Along with other polyphenols, including resveratrol and tea polyphenols, quercetin has been proposed for IBD and colitis treatment to attenuate inflammation, as they can regulate various cytokines and chemokines expression, as well as inhibit nuclear factor-kappa B (NF-κB) transcription (a vital factor for the initiation of an inflammatory response) [150]. They modulated the NF-κB activation cascade by blocking IKK activation, which inhibits IκBs phosphorylation or degradation and the nuclear translocation of p50/p65 [151]. Dietary quercetin is a promising strategy for IBD amelioration following its capacity to restore intestinal immune hemostasis and enteric commensal microflora balance via modulating the Nrf2/HO-1 pathway [152]. In colon tissues, dietary quercetin (30 mg/kg) attenuated colitis severity by modifying gut microbiota population (increased *Bacteroides*, *Bifidobacterium*, *Lactobacillus*, and *Clostridia*, with reduction in *Fusobacterium* and *Enterococcus* populations) and inflammatory cytokines milieu (promoted IL-10 production, but reduced pro-inflammatory cytokines, such as IL-17, TNF-α, and IL-6) [125]. However, there exist several inflammatory bowel disease models applied for research, and they differ in their immune and pathophysiological characteristics, which may give rise to discrepancies. For instance, it had been explained that quercetin was unable to ameliorate DSS-induced colitis due to its absorption by glycosides in the small intestine. Therefore, quercetin prodrugs, such as rutin and quercitrin, were shown to facilitate quercetin’s release in the colon [153]. Quercetin’s glycoside, rutin, has also been demonstrated as a therapeutic agent against IBD, since it can serve as a prodrug for quercetin delivery near IBD sites, alongside its antioxidative and anti-inflammatory effects [154].

(b) Nitric oxide regulation

l-citrulline, as a functional amino acid with biological functions including cellular metabolism and organ function [37], is also an efficient precursor for NO synthesis [43]. NO is involved in multiple physiological processes in the GIT, particularly the regulation of gastrointestinal motility, gastroprotection, and mucosal blood flow [35]. However, higher NO concentrations are toxic since NO reacts with superoxide anion to form peroxynitrite, which in turn promotes cytotoxicity, inflammation, oxidative stress, and increased intestinal epithelial permeability [155], key features of IBD. Studies suggest that IBD is associated with elevated iNOS activity, and as such, increased NO production [35]. Circulating levels of nitrite and nitrate (NO metabolites) were increased and positively associated with intestinal iNOS activity during ulcerative colitis and Crohn’s diseases [35,156]. Contrarily, in another report, animal models of experimental IBD showed that both constitutive and inducible NO production were beneficial during acute colitis, although prolonged upregulation of NO was detrimental [157]. Using Argininosuccinate lyase (ASL) knockout mice induced with colitis, it was revealed that enterocytes-derived NO alleviated colitis by reducing macrophage infiltration and tissue damage [158]. However, NO derived from the immune cells was responsible for macrophage activation and increased severity of inflammation [158]. This finding reveals that the specific intracellular source of NO may be implicated in the NO positive and/or negative role during IBD pathogenesis. Interesting is the capacity for citrulline to not only act as an arginine precursor for NO production, but also the question of citrulline’s involvement in NO regulation during biological processes. Impairment in citrulline availability and de novo arginine synthesis reduced NO production but was restored with citrulline supplementation [159]. Thus, citrulline is implicated in the regulation of NO production for homeostasis, as against NO overproduction and toxicity [160].

NO plays an important role as an endogenous vasodilator and promotes endothelial functions in the gastrointestinal system [161]. Studies have shown that quercetin augments NO production and vasodilation via eNOS activation [162], eliminating endothelial dysfunction [163]. In addition, quercetin was shown to induce vasodilation by enhancing NO synthesis and promoting intracellular calcium-activated potassium channels [164]. Quercetin exerted endothelium-dependent vasodilatations via invoking sustained nitric oxide release in mesenteric vascular beds isolated from rats [165]. Quercetin also restored the intravascular homeostasis and endothelial functions, by attenuating excess NO production induced by ATP, decreased intracellular calcium flux, and eNOS activity in vascular endothelial cells [166]. Further, quercetin reversed the endothelial damage arising from excessive NO by attenuating nitrification stress and protecting the endothelial cells [164]. In mice IBD models, quercetin monoglycosides were shown to counteract increased serum NO and oxidative stress resulting from DSS-induced colitis [134]. In euglycemic and diabetic rats, quercetin supplementation increased the bioavailability of NO in the jejunum [167]. Furthermore, co-administration of quercetin and/or L-arginine (each 200 mg/kg body weight) provided protection against cardiotoxicity [168] and hepatotoxicity [169,170] in rat models.

(c) Anti-oxidative functions

Reactive oxygen species (ROS) generation in the gut has been linked with several inflammatory disorders. Excessive levels of ROS lead to cellular damage, ultimately disrupting the intestinal barrier, increasing gut permeability and tissue damage [171]. Increased oxidative stress is associated with mucosal inflammation during ulcerative colitis [134,171], and an overproduction of ROS may contribute to the progression of this disorder. Using a DSS-induced colitis mice model, treatment with quercetin aglycone alone or quercetin aglycone with monoglycosides counteracted both inflammatory response and oxidative stress, by lowering the malondialdehyde (MDA), reduced glutathione (GSH), serum nitrate (NO), and myeloperoxidase (MPO) concentrations [134]. Under oxidative stress conditions, citrulline and quercetin would be useful in combating ROS and altering the redox status of the gut. Citrulline has been widely known for its antioxidant properties, which has been attributed either to the direct effects of citrulline and/or indirect effects via NO production [160,172]. Citrulline directly mediated antioxidant defense by influencing the enzyme activities of catalase, superoxide dismutase (SOD), and the total antioxidant capacity, but decreased MDA contents, abolishing the degree of lipid peroxidation [43]. l–citrulline was shown to protect the gastric mucosa during ethanol-induced oxidative stress by recovering SOD, and GSH-Px activities approximately to the control levels [19]. Alongside this, as a potent antioxidant, quercetin acts to maintain endogenous antioxidant defenses, and scavenges reactive oxygen species (ROS) via suppressing the synthesis of lipid peroxy radicals [173], and chelating of metal ion [174]. Quercetin-induced antioxidant effects through activating the Nrf2/NRF1 transcription pathway, which upregulates the expression of peroxiredoxins (PRDX3 and PRDX5), an antioxidant family responsible for catalyzing hydrogen peroxide reduction [175]. Nrf2 transcription affects downstream targets such as catalase, superoxide dismutase 1, glutathione peroxidase 2, heme oxygenase-1 (HO-1), and thioredoxin genes, ameliorating oxidative stress [121]. Further, in vitro cell culture with quercetin showed upregulated phosphorylation for protein kinase B (Akt) and extracellular signal-regulated kinase 1/2 (ERK1/2), with or without hydrogen peroxide treatment, where Akt and ERK1/2 induction plays protective roles against oxidative stress [176]. Moreover, during intestinal oxidative stress, quercetin restores the normal redox status, in addition to promoting intestinal calcium absorption via glutathione and the glutathione-dependent enzymes system [32]. These findings suggest that both citrulline and quercetin treatment promote the antioxidant machinery by elevating antioxidant enzyme levels, and reduce the levels of ROS and lipid peroxidation products. We consider this particularly useful in conditions such as heat stress, which is closely associated with oxidative stress [177], since heat stress tends to influences the metabolic rate, antioxidant defenses, immune function, inflammatory status, gut functions, reproductive ability, organ functioning, and in severe cases, the lifespan of the animals [178,179,180].

(d) In vitro effects of citrulline and quercetin on intestinal cell integrity

Studies have reported on the in vitro effects exerted by citrulline and quercetin on the cellular intestinal integrity. For instance, Chapman et al. [181] reported that arginine (synthesized from citrulline) and citrulline (key precursor of arginine) protected the intestinal cell monolayer tight junctions from hypoxia-induced injury in piglets. They further demonstrated that NO played a major role in the protective effects of arginine and citrulline during intestinal epithelial hypoxia [181]. This suggests the beneficial role of NO in the maintenance of intestinal barrier functions. During arginine supplementation, NO was implicated in cell migration in razor-injured porcine intestinal epithelial cell monolayers [182]. Further, NO derived from iNOS following arginine and citrulline supplementation was involved in re-epithelialization of laser-wounded renal tubular cell monolayers and deoxycholate-injured porcine ileal mucosa [183,184]. It is known that constitutive and iNOS activity is present in the intestinal epithelia of several species, thus facilitating the protective effects of NO donors such as arginine and citrulline [15,182]. Reports indicated that arginine and citrulline were involved in decreasing inulin flux across hypoxic monolayers and qualitatively preserved tight junction proteins [181]. Taken together, these findings showed that arginine and citrulline, via a mechanism dependent on NO donation, protects the intestinal epithelial integrity. Inflammation in porcine intestinal tract affects absorption of nutrients, reduces growth performance, and also decreases immunity, leading to pathogenic microorganisms infections in animals [185]. Intestinal porcine enterocyte cells-jejunum2 (IPEC-J2) are normal cells derived from the jejunal epithelial cells of the piglet with good biological characteristics of intestinal epithelial cells [186,187]. Lipopolysaccharide (LPS), an endotoxin, and other related toxic substances such as aflatoxin, can trigger inflammatory response in intestinal cells such as IPEC-J2 by stimulating the expression of inflammatory cytokines including IL-6 and IL-8 [188]. Quercetin possesses anti-inflammatory potential that can be expressed on different cell types, both in animal and human models [189,190]. Chen et al. [191] conducted an in vitro experiment to determine whether or not quercetin had the potential to inhibit inflammation in the small intestine of pigs by initially pretreating IPEC-J2 with quercetin, and then LPS. It was confirmed that pre-treatment of quercetin showed protective effects on the intestinal porcine enterocyte cells and inhibited porcine intestinal inflammation induced by LPS. Quercetin promotes mast cell stability, gastrointestinal cytoprotection, and also modulates gut immunity [190,192].

Therefore, since quercetin plays several biological roles just as l–citrulline with regards to promoting gut health, we propose that there would exist synergistic, beneficial interactions when these two bio-active substances are utilized together, to promote gut health. Thus, a citrulline + quercetin combination may assist in ameliorating intestinal disorders such as IBD pathogenesis, via restoring the intestinal host-microbiota relationship, immunological homeostasis, modulation of pro-and anti-inflammatory cytokines milieu, and enhancing the antioxidant properties of intestinal tissues. Studies have also explored the effectiveness of using quercetin or citrulline in combination with other components, mainly to harness their beneficial effects. Supplementation of citrulline with *Lactobacillus helveticus* (a bacteria strain that can catabolize arginine/citrulline for cell growth) exhibited synergistic effects in promoting NO production and improving intestinal epithelial barrier functions [98]. In a study investigating the effects of magnolol (an active ingredient utilized in traditional Chinese medicine) in L-arginine-induced gastrointestinal motility disorder, it was reported that magnolol alleviated gut disorder via reducing NO production, and its relaxing properties on GIT [193]. Another study employing the combination of dasatinib and quercetin as senolytic drugs in aged mice revealed that their long-term treatment reduced senescence cells, reduced inflammatory markers, and altered metabolic signatures in the intestines, thus improving gut health [194]. Additionally, studies have combined polyphenols such as resveratrol and quercetin widely for their health benefits, and their role in modulating the gut microbial ecosystem [2,28]. Combination of rice bran and quercetin altered gut microbiome composition and metabolites, such that there was a significant shift to enrich the proliferation of beneficial bacteria, while the population of opportunistic pathogens in the gut was reduced [195]. Quercetin and catechin combination was reported as a potential therapy for alleviating excessive adipose tissue inflammation, improving metabolic parameters related to insulin sensitivity, and regulating cell redox status to exert anti-inflammatory actions [196]. In addition, quercetin elicited synergistic roles together with vitamin E to promote anti-inflammation, anti-apoptosis, and immunity in aged hens [197,198].

Presently, studies investigating the interaction between citrulline and quercetin are unavailable, and this present review intends to direct research focus towards their potentials in promoting gut health. Therefore, since both citrulline and quercetin are natural bioactive compounds found majorly in a variety of foods, such as citrus, vegetables, and beverages, we suggest the possible beneficial effects of these two metabolites, owing to several studies elucidating on their diverse biological properties, including anti-inflammatory, antioxidant, and gut-modulating effects, as well as other bioactivities.

## 5. Knowledge Gaps and Future Perspectives

Several studies have revealed citrulline as an efficacious substance with anti-inflammatory, anti-oxidative properties, and a high capability to mediate gut functions. The ability of citrulline to serve as a potent arginine precursor allows for its potential application under conditions of arginine deficiency, such as in inflammatory states [9]. However, most of the studies conducted on citrulline’s effects have been linked to its ability as an arginine precursor and NO-dependent mechanisms. Thus, there is a paucity of research on the direct effects of citrulline and interactions that may be involved, warranting further investigations.

With citrulline’s role as a gut biomarker, and a functional metabolite of the gut, as well as the numerous reports supporting quercetin’s role in gut microbiota modulation, we suggest that synergistic effects may exist when these two substances are co-utilized, considering they have similar attributes of antioxidative, anti-inflammatory, and immune-regulatory roles in the gut, especially under pathophysiological conditions. Importantly, the effectiveness of quercetin in alleviating colitis and IBD treatment is impeded due to its rapid metabolism and primary absorption in the upper gastrointestinal tract, as such limiting its transit and absorption in the lower gastrointestinal tract [199]. This problem is counteracted by supplying quercetin in its glycosylated form, commonly as rutin, which allows for passage through the epithelial cells, and conversion by the gut microbiota to derive the bioactive effects of quercetin [199]. Moreover, in the gut, citrulline is easily absorbed by the gut from the enterocytes lumen such that oral supplementation with citrulline is more efficient than with arginine supplementation to supply arginine [37]. An interesting discovery would be whether citrulline can interact with quercetin to facilitate its absorption in the gut or otherwise. To the best of our knowledge, there are currently no studies that have examined the mutual effects of citrulline and quercetin on gut functions, and the primary aim of this review is to elucidate the benefits of each bioactive substance, while drawing attention to the potential synergistic effects that could be derived when co-utilized. Therefore, investigations to uncover their areas of synergy and antagonism are warranted, especially considering their bioavailability from various plant sources. Prospective studies are necessary to assess the specificity and sensitivity of these dual supplements as possible modulators of intestinal inflammation. Further, it would be interesting to unveil other biological roles for which they may be utilized (apart from the areas covered in this review), during health and pathological states. Furthermore, gut microbiota studies have been considered the novel approach for understanding gut health problems, and thus, the possible benefits of citrulline and quercetin for gut health should be explored through microbiome evaluation, and the association of the omics analysis at the gene, transcripts, proteins, and metabolite levels, to ascertain the biological effects of citrulline + quercetin in microbiota interactions.

## 6. Conclusions

This review has given a recent account of citrulline and quercetin in modulating the intestinal immunity, redox status, and inflammatory conditions in the gut. Studies have demonstrated both citrulline and quercetin as potent bioactive substances, eliciting various beneficial effects on gut functioning and health. Thus, this comprehensive review outlined the major biological roles played by both citrulline and quercetin on gut integrity and microbial composition, and further elucidated the synergistic roles that would exist in utilizing both citrulline and quercetin in health and disease states. Therefore, citrulline and quercetin co-treatment could provide the next novel therapeutic outlook needed to promote gut functions and host health under pathophysiological states.

## Figures and Tables

**Table 1:** Summarized roles of citrulline in promoting intestinal immune response and gut health in human and animal models.

Subject	Design	Main Findings	References
Wistar rats; Beagle dogs; Cynomolgus monkeys	Intestinal toxicity was induced using oncological drug candidates.	In treated animals, a > 50% decrease in plasma l-citrulline levels strongly correlated with histopathological findings in the small intestine such as single-cell necrosis and mucosa atrophy, intestinal crypt necrosis, villus atrophy, enterocyte loss, and clinical signs (bloody feces and diarrhea), indicating l-citrulline as a small intestine biomarker.	[104]
Dogs (5 males/5 females per group)	Oral doses of 0.75, 1.5, and 3 mg/kg/d of MS-229 over 4 weeks to induce small intestinal toxicity.	A dose- and exposure-dependent decrease in plasma citrulline was correlated with pathological findings in the small intestine.	[105]
Preterm infants	Plasma citrulline levels were measured during the first 48 h after necrotizing enterocolitis onset.	Plasma citrulline decreased in the first 48 h suggesting ongoing intestinal injury, thus plasma citrulline measurement may provide an indication for intestinal recovery rate during the first 24 h after NEC onset.	[106]
Male Wistar rats (*n* = 46; 230–250 g)	Varying citrulline levels were administered as 0.5,1, 2.5, 5 g/kg/d citrulline.	The jejunum weight was significantly positively correlated with plasma citrulline, suggesting a dose-dependent intestinal adaptation in gut resected rats.	[107]
In vitro analysis using IPEC-J2 cells	Citrulline (2 mM) and *Lactobacillus helveticus* ASCC 511 were co-treated to IPEC-J2 cells.	*Lactobacillus helveticus* and citrulline exhibited synergistic effects against adhesion of pathogenic bacteria, *Escherichia coli;* stimulated nitric oxide; improved transepithelial electrical resistance; and stimulated tight junction proteins expression, thus, promoting intestinal health.	[98]
Female C57BL/6J mice	Mice were induced non-alcoholic steatohepatitis using fat-, fructose-, and cholesterol-rich diet followed by +/− 2.5 g l -citrulline/kg body weight.	l-citrulline alleviated non-alcoholic fatty liver disease progression via attenuation of bacterial endotoxin translocation and the loss of tight junction proteins in small intestinal tissue.	[108]
Human model	Randomized, double-blind crossover study, 10 men cycled for 60 min at 70% of their maximum workload after l-citrulline (10 g) or placebo (l-alanine) intake.	Pre-exercise l-citrulline intake prevented splanchnic hypoperfusion-induced intestinal compromise by preserving splanchnic perfusion and attenuated intestinal injury during exercise probably by enhancing arginine availability.	[109]
Mice model	Mice undergoing intestinal obstruction were divided into three groups: sham, intestinal obstruction, and citrulline group receiving a diet containing 0.6% citrulline.	Citrulline pretreatment preserved barrier integrity and modulated immune response via decreasing intestinal permeability and bacterial translocation, whereas it preserved the ileum mucosa and increased secretory IgA concentration.	[110]
Male Wistar rats (*n* = 15)	Ulcerative colitis was established in rats and citrulline was administered intragastrically for 7 d.	Citrulline provided protective effects by lowering the peripheral blood monocytes, the infiltration of CD68-positive monocytes, and the concentrations of MCP-1, IL-6, and IL-17A in the colon tissues of effects in ulcerative colitis rats.	[111]
Adult male Sprague–Dawley rats (180–220 g)	l-citrulline (300, 600, and 900 mg/kg body weight) was administered to rats having ethanol-induced gastric ulcer in rats.	l-citrulline elicited gastro-protective effects by attenuating gastric lesions, prevented oxidative damage, decreased nitric oxide content and increased the myeloperoxidase activity	[19]
Male Wistar rats (*n* = 24, 220–230 g)	Rats were assigned to either citrulline, arginine, control, or sham groups. The sham group underwent transection and other groups had an 80% resection of the small intestine.	Citrulline increased arginine levels and improved nitrogen balance after massive intestinal resection.	[112]
Adult male Sprague–Dawley rats (200–240 g)	l-citrulline (300, 600, and 900 mg/kg) was pretreated to ischemia/reperfused rats.	l-citrulline reduced the gastric mucosal lesion, prevented the production of lipid peroxidation, and inhibited the increase in myeloperoxidase activity.	[113]
Male C57/Bl6 mice (*n* = 65; 26–28.5 g)	Mice received intravenous infusion of endotoxin (LPS, 0.4 µg/g bodyweight per h) combined with either l-citrulline (6.25 mg/h), l-arginine (6.25 mg/h), or l-alanine (12.5 mg/h).	During endotoxemia, l-citrulline supplementation reduced intestinal microcirculatory dysfunction and increased intracellular NO production via increasing plasma and tissue concentrations of arginine and citrulline, and restored intracellular NO production in the intestine. Jejunal tissues in the l-citrulline group showed an increase in degree of phosphorylation of eNOS phosphorylation and decreased iNOS protein level.	[45]
Swiss male mice (6 weeks old)	Mice received supplemented citrulline or alanine in the drinking water for 10 d (1 g/kg/d) and on the seventh day, the animals were injected intraperitoneally with a single dose of phosphate-buffered saline (PBS) or 5-fluorouracil (200 mg/kg) for the induction of mucositis.	Citrulline administration contributed to a partial recovery of the mucosal architecture in mucositis-induced mice. There was an intermediate reduction in the histopathologic score, and functional intestinal permeability was partially rescued by citrulline treatment. Citrulline attenuated mucosal damage by reducing the size of the injured areas and decreased intestinal permeability in mucositis mice.	[114]

**Table 2:** Summarized roles of quercetin in enhancing intestinal immunity and gut health in human and animal models.

Subject	Design	Main Findings	References
The LDL receptor-deficient mouse C57BL/6 mice (90 days old; (24.76 ± 0.37 g)	Mice were randomly assigned to either the quercetin treatment (100µg/d; *n* = 12) or the control group (*n* = 12) and fed regular chow diet for 4 weeks, followed by a high-fat diet until 12 weeks.	Quercetin treatment to high-fat-diet-fed mice attenuated atherosclerotic lesions, elicited protective effects against immune/inflammatory responses and oxidative stress, and decreased intestinal lipid levels. Additionally, quercetin altered the gut microbiota composition by decreasing the abundance of *Verrocomicrobia* but increased microbiome diversity and the abundances of *Actinobacteria*, *Cyanobacteria*, and *Firmicutes.* Quercetin reduced the lipid level, areas of atherosclerotic lesions and sizes of plaques.	[124]
C57BL/6 mice	Dietary quercetin (30 mg/kg) was supplemented to a *Citrobacter rodentium*-induced colitis mouse model for 2 weeks.	Quercetin alleviated *Citrobacter rodentium*-induced colitis by suppressing pro-inflammatory cytokines production and modified the gut microbiota by increasing *Bacteroides*, *Bifidobacterium*, *Lactobacillus*, and *Clostridia* populations but reduced *Fusobacterium* and *Enterococcus* spp.	[125]
Broiler chickens (*n* = 240)	Chickens were randomized into four groups: saline-challenged; LPS-challenged; and LPS-treated broiler chickens, fed either 200 or 500 mg/kg of quercetin.	Quercetin alleviated LPS-induced oxidative stress via the MAPK/Nrf2 signaling in the intestines of chickens. Quercetin alleviated LPS-induced decrease in duodenal, jejunal, and illeal villus height and increased the crypt depth of these regions. Further, quercetin inhibited LPS-induced jejunal oxidative stress and relieved jejunal mitochondria damage.	[126]
Finishing pigs ((Large White × Landrace); *n* = 170; initial body weight of 72 ± 4 kg)	Pigs were randomly assigned to either a control group fed basal diet or treatment group consuming the same diet supplemented with 25 mg/kg feed quercetin, and after a 4-week period, pigs were transported for 5 h.	Quercetin-supplementation improved intestinal health and alleviated intestinal injury during transport through decreased serum endotoxin levels, lowered intestinal ROS and MDA, and lowered jejunal inflammatory cytokines expression, but increased jejunum villi height and upregulated the mRNA expression of occludin and zonula occudens-1 in the jejunum.	[127]
Male Wistar rats (8 weeks old; 250 ± 20 g)	Post-inflammatory irritable bowel syndrome (PI-IBS) model rats were administered quercetin by gavage at doses of 5, 10, and 20 mg/kg for 14 d.	Quercetin elicited an analgesic effect on PI-IBS and decreased the visceral pain threshold of PI-IBS rats, and the abdominal motor response to colon distension was markedly increased. Quercetin also reduced the colonic expression of genes responsible for enteroendocrine cell differentiation.	[128]
Rats	Rats were grouped as osteoarthritis-induced model, quercetin-treated, and control groups. Quercetin group received daily intragastric administration (100 mg/kg/d, i.g.) from day 1 to day 28.	Quercetin partially abrogated intestinal flora disorder and reversed fecal metabolite abnormalities. Diversity in the gut microbiota was decreased after quercetin treatment and at the genus level, *Lactobacillus* was increased whereas, unidentified *Ruminococcaceae* was decreased.	[129]
Ross 308 chicks (*n* = 128 chicks; 41 gm/chick)	Quercetin was fed to groups of broiler chickens at concentrations of 200, 400, and 800 ppm, and a control group was supplemented with a basal diet.	Dietary quercetin improved the gut microbiota environment by decreasing total coliforms and *Clostridium perfringens* population but increased the *Lactobacillus* counts. Further, the intestinal mRNA expression of intestinal Cu/Zn-superoxide dismutase, glutathione peroxidase, and nutritional transporters was upregulated in quercetin-supplemented groups.	[130]
C57BL/6J mice	Monosodium glutamate (MSG)-treated mice were randomly divided into two groups: MSG group and quercetin group (5 mg/kg quercetin) administrated by gavage at a dose of 100 µL/10 g/body weight (BW)/ d for 6 weeks.	Dietary quercetin attenuated MSG-induced gut microbiota dysbiosis and improved intestinal barrier function. Quercetin reversed MSG-induced elevation in *Firmicutes* abundance and decreased the *Firmicutes*/*Bacteroidetes* ratio. Further, *Lachnospiraceae* and *Ruminicoccaceae* abundance was reduced. Colon damage was recovered and Muc2 and ZO-1 expression was upregulated after quercetin treatment.	[131]
Wistar rats (*n* = 23)	Wistar rats were randomized into four groups fed a high-fat sucrose diet supplemented or not with trans-resveratrol (15 mg/kg body weight (BW)/d), quercetin (30 mg/kg BW/d), or a combination of both polyphenols.	Quercetin supplementation eliminated gut dysbiosis by attenuating *Firmicutes*/*Bacteroidetes* ratio and inhibited the growth of bacterial species associated to diet-induced obesity (*Erysipelotrichaceae*, *Bacillus*, *Eubacterium cylindroides*).	[28]
Male C57BL/6J mice (7 weeks old)	Mice were challenged with high-fat diet (HFD) supplemented or not with quercetin (0.05% (wt/wt) aglycone quercetin) for 16 weeks.	Quercetin alleviated obesity-associated NAFLD via its anti-inflammatory, antioxidant, and prebiotic integrative response. Quercetin reverted gut microbiota imbalance and related endotoxemia-mediated TLR-4 pathway induction, with subsequent inhibition of inflammasome response and reticulum stress pathway activation.	[123]
Kunming male mice (*n* = 36; 18–20 g)	Mice were administrated 0.5 mL/d antibiotics cocktail intragastrically for 7 d to induce gut dysbiosis. Quercetin-treated mice were fed AIN-93G diet containing 0.2% quercetin for 10 d.	Quercetin supplementation combated gut dysbiosis since it recovered intestinal barrier function and improved the diversity of the gut bacterial community in antibiotic-treated mice. Intestinal villi length and mucosal thickness were increased and butyrate production was enhanced in quercetin-treated mice.	[120]
Sprague–Dawley rat (6 weeks old; male; 160−200 g)	Quercetin (50 mg/kg/d) was dissolved in distilled water and administered daily by gavage at 10 mL/kg for 12 weeks to streptozotocin (STZ)-induced diabetic peripheral neuropathy (DPN) rats.	Quercetin exerted a neuroprotective effect and modulated gut microbiota associated with DPN phenotypes and ROS production in STZ-induced DPN rats. Quercetin rescued gut dysbiosis by decreasing four potential pathogenic species and enriching two prebiotic species associated with DPN phenotypes and ROS production.	[132]
